# Investigation of organic impurity and its occurrence in industrial waste salt produced by physicochemical process

**DOI:** 10.1371/journal.pone.0256101

**Published:** 2021-08-20

**Authors:** Zongwen Zhao, Wenbin Xu, Zhongbing Wang, Weining Qin, Jie Lei, Xinglin Guo, Jiang Long

**Affiliations:** 1 School of Metallurgy & Environment, Central South University, Changsha, Hunan, China; 2 Postdoctoral Mobile Station of Central South University, Changsha, Hunan, China; 3 Dongjiang Environmental Co., Ltd., Shenzhen, Guangdong, China; 4 Green Eco-Manufacture Co., Ltd., Shenzhen, Guangdong, China; Tsinghua University, CHINA

## Abstract

Industrial waste salt is classified as hazardous waste to the environment. The organic impurity and its occurrence in industrial waste salt affect the salt resource utilization. In this paper, composition quantitative analysis, XRD, TG-DSC, SEM/FIB-SEM coupled with EDS, FTIR, XPS and GC-Ms were chosen to investigate the organic impurity and its occurrence in industrial waste salt. The organic impurities owe small proportion (1.77%) in the specimen and exhibit weak thermal stability within the temperature of 600°C. A clear definition of organic impurity, including 11 kinds of organic compounds, including aldehyde, benzene and its derivatives etc., were detected in the industrial waste salt. These organic impurities, owing (C-O/C-O-C, C-OH/C = O, C–C/CH_*x*_/C = C etc.)-containing function group substance, are mainly distributed both on the surface and inside of the salt particles. Meanwhile, the organic substance may combine with metal cations (Ni^2+^, Mg^2+^, Cu^2+^ etc.) through functional groups, such as hydroxide, carbonyl etc., which increases its stability in the industrial waste salt. These findings provide comprehensive information for the resource utilization of industrial waste salt from chemical industry etc.

## 1. Introduction

Large quantities of high salinity wastewater are inevitably produced in these industrial activities that include food-processing industry, leather industry, petroleum industry etc. [1, 2]. The typical characteristic for the high salinity wastewater is the containing of toxic and harmful substances [3]. For example, coal chemical high salinity wastewater contains heavy metals (Hg^2+^, Pb^2+^ and Cd^2+^) [4]; metallurgy, and surface finishing industries high-salinity wastewater contains Ni (II) [5]; pickling and elutriation wastewater is characterized by high organic load, high level of nitrogen [6]; mustard tuber wastewater is rich in ammonia (NH_4_^+^) and sulfate (SO_4_^2−^) [7]. Therefore, the direct discharge of untreated high-salinity wastewater can bring serious environmental damage to aquatic, terrestrial, and wetland ecosystems [3, 8, 9]. Besides, the toxic substances in the salinity wastewater have strong inhibition on microbial metabolism, nitrification and denitrification performances [10, 11]. Thus, it is believed that biotechnology is not suitable for treating high salinity wastewater; while physico-chemical techniques (including evaporation, ion exchange, and membrane processes etc.) can be chosen to treat most of high salinity wastewater [2] Of various physico-chemical techniques, evaporation crystallization technology is widely used to achieve “zero discharge” of high-salinity wastewater by transforming high salinity wastewater into mixed solid salt [12, 13]. It has been reported that the typical projects of high-salt wastewater treatment methods in China, 60% are evaporation crystallization [12]. The evaporation crystallization technology includes concentration pretreatment, evaporation concentration and crystallization. The pretreatment process mainly includes filtration, pH adjustment, coagulation and defluorination etc., which can inhibit the scaling in the following operation and lower the operation cost. The evaporation concentration, which is mainly conducted by multistage evaporation or MVR, is aimed to evaporate water and brings the salt to saturation. While the crystallization section, including evaporation crystallization and cooling crystallization, is to separate the salt from the concentrated solution [14, 15].

The evaporation crystallization technology can realize the separation of salt from wastewater and make the water reuse. However, the application of this technology will inevitable produce a large amount of mixed industrial waste salt. It has been reported that the annual output of industrial waste salt is around 20 million tons in China [16]. The industrial waste salt produced by high salinity wastewater owes the following typical characteristics. For one thing, the organic impurity owes non-volatile and the content is up to 25% [17]. For another, the organic impurity has complicated chemical composition. Lastly, the organic impurity may combine with the heavy melts. These characteristics make industrial waste salt difficult for recycling. Besides, according to the latest National Hazardous Waste List (2021 edition) of China, most of the industrial waste salt are classified as hazardous waste and need special treatment [18]. In addition, the market capacity for reusing industrial waste salt is limited, which results in limited economic interest for enterprises to recovery the salt resources. Hence, landfill is often chosen to dispose these industrial waste salt. However, with the strengthening of environmental regulation, landfill disposal of industrial waste salt in flexible landfill sites is almost forbidden. Employing rigid landfill method to dispose industrial waste salt must meet the requirements of standard for pollution control on the hazardous waste landfill (GB 18598–2019) in China [19]. Therefore, it is urgent to develop industrial waste salt recycle technology and realize the separation of useful salt from the waste [20].

The premise to reach this goal is to have a good knowledge of the organic impurity and its occurrence in industrial waste salt, which plays an important role in the development of industrial waste salt recycling technology. In this paper, a typical industrial waste salt was chosen from evaporation crystallization process as the subject. Composition quantitative analysis, X-ray diffractometer (XRD), thermogravimetric-differential scanning calorimeter (TG-DSC), scanning electron microscopy/focused ion beam scanning electron microscopy (SEM/FIB-SEM) combined with energy dispersive spectrometer (EDS), fourier transform infrared spectroscopy (FTIR), X-ray photoelectron spectroscopy (XPS) and gas chromatograph-mass spectrometry (GC-MS) were jointly used to study the organic impurity and its occurrence. These findings can be helpful to the development of industrial waste salt recycle technology.

## 2. Process description and study method

### 2.1 Process description

**Fig 1:**
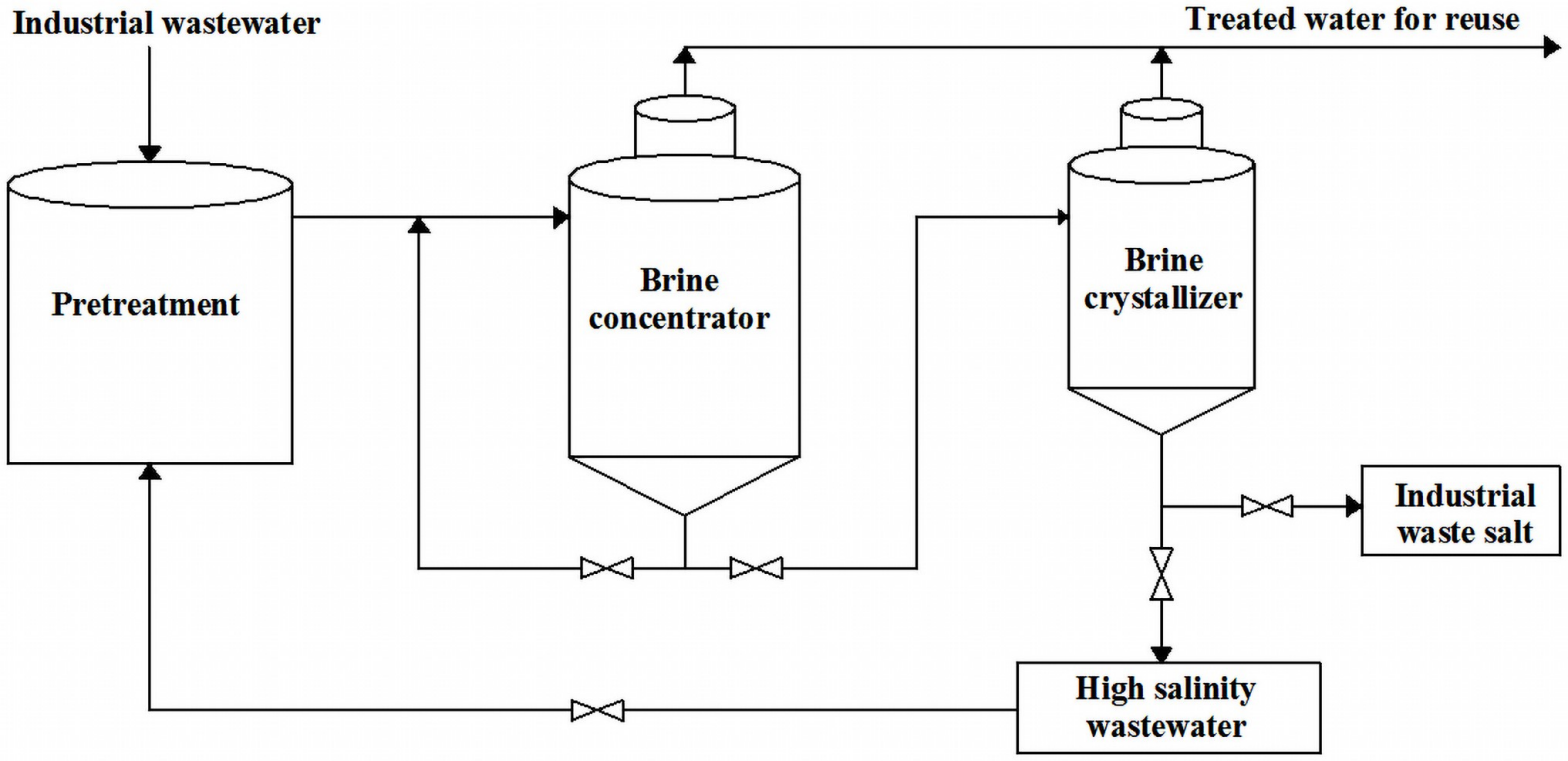
The evaporation crystallization process for the production of the industrial waste salt.

The typical evaporation crystallization process is presented in Fig 1. The high salinity wastewater produced by different industries is mixed and pretreated by coagulation, defluorination, and descaling etc. Subsequently, the pretreated high salinity wastewater enters into the evaporation concentrator, which makes the salt reach the saturation. Then, the saturation liquid is pumped to the crystallization section to crystallize. Finally, the crystalline salt is separated by centrifugation. The typical crystalline salt i.e. industrial waste salt was collected for the following study.

### 2.2 Composition determination

The TOC was analyzed by combustion oxidation-non-dispersive infrared absorption method (NY/T 52–1987); Nitrate was analyzed by ultraviolet spectrophotometry (HJ/T346-2007); Sulphate was analyzed by gravimetric method; Ammonia nitrogen was determined by distillation titration method (GB/T2946-2018); Chloride ion was determined by silver nitrate titration method (GB/T-11896-1989); The total phosphorus was determined by ammonium molybdate spectrophotometric method (GB/T 11893–1989); The remaining metal elements were determined by ICP-AES, AAS etc.

### 2.3 Characterization analysis

The crystallographic composition was identified by XRD (Empyrean 2, Malvern Panalytical, Netherlands) in 2θ ranging from 10° to 80°; The microstructure was observed by SEM/FIB-SEM (TESCAN MIRA3 XMH and FEI Helios Nanolab 600i) equipped with an EDS system (QUANTAX 400, Bruker). FTIR was collected in the range of 400–4000 cm^-1^ using a BRUKER, VERTEX70 spectrometer at 4 cm^-1^ resolution using the KBr pellet technique; XPS experiments were carried out on a Thermo Fisher Nexsa using an Al K-a X-ray source. All spectra were calibrated with graphitic carbon as the reference at the binding energy (BE) of 284.8 eV; The comprehensive thermal behavior of the specimen in the temperature ranging from room temperature to 1000°C was analyzed by a TG-DSC apparatus (NETZSCH STA 449 C) in argon flowing at the heating rate of 10°C/min, and a lid was putted on the crucible during the measuring. The Agilent GC-MS (GC7890A-MS5975C) was used to determine the relative amount of the volatile organic compounds possibly existed in the industrial waste salt.

## 3. Results and discussions

### 3.1 Characteristics of the industrial waste salt

#### 3.1.1 Chemical compositions

The chemical compositions of the industrial waste salt are presented in Table 1. It can be seen that the main chemical compositions of the industrial waste salt are Cl^-^ (32%), SO_4_^2-^ (19.74%), Na^+^ (28.3%), which implies the main chemical phases in the industrial waste salt are NaCl and Na_2_SO_4_. The moisture content is around 5% in the specimen and the remaining chemical compositions are ascribed to impurities. Varieties of salinity wastewater are mixed in the evaporation crystallization process and therefore different kinds of impurities are all introduced into the industrial waste salt, which can be classified into two categories i.e. inorganic and organic impurities. The inorganic impurities include Ni^2+^, Mg^2+^, Cu^2+^ etc. cationic and F^-^, NO_3_^-^ etc. anionic. The organic impurities, which represent by TOC, owe small mass proportion (1.77%) in the industrial waste salt. In addition, these organic impurities have undergone high temperature evaporation process, which owe the property of non-volatile. These impurities can deteriorate the salt quality and need deep treatment in the process of salt resource utilization.

**Table 1:** The chemical composition of the industrial waste salt.

Chemical composition	TOC	Moisture	T-P	N-NH_4_	Cl^-^	NO_3_^-^	SO_4_^2-^	F^-^
Content (wt %)	1.77	5.22	0.77	0.69	32.00	5.02	19.74	0.28
Chemical composition	Na^+^	Ca^2+^	K^+^	Fe^3+^	Cu^2+^	Mg^2+^	Ni^2+^	others
Content (wt %)	28.30	1.35	0.57	0.06	0.38	0.40	0.14	3.31

#### 3.1.2 Phase compositions

**Fig 2:**
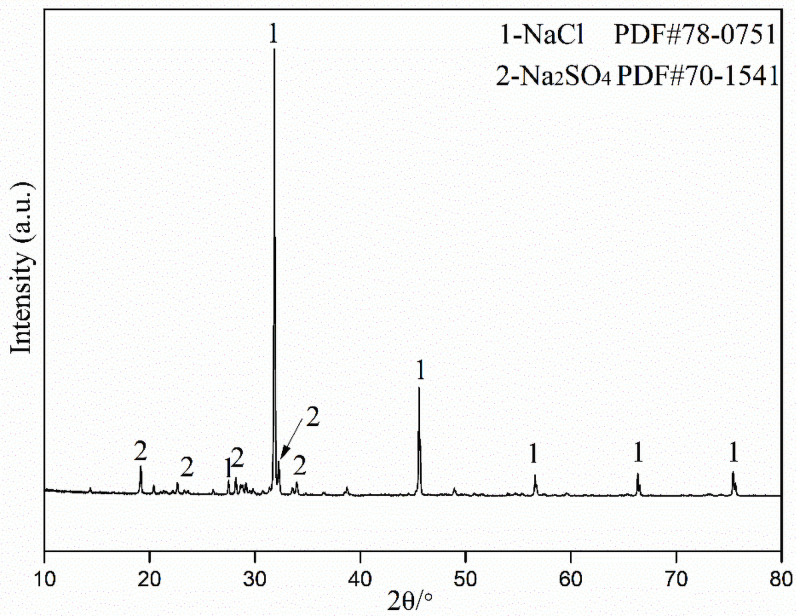
XRD pattern of the industrial waste salt.

The XRD measurement has been submitted to study the crystal phases in the industrial waste salt and the results are presented in Fig 2.

As can be seen, NaCl (PDF#78–0751) and Na_2_SO_4_ (PDF#70–1541) are the main crystal phases identified in the XRD pattern. In addition, due to the small content of organic impurity and other inorganic salts, their characteristic peaks may cover up by the background noise. Therefore, it is necessary to use other techniques to further study the occurrence of the impurities in the industrial waste salt.

#### 3.1.3 Thermal behavior analysis

**Fig 3:**
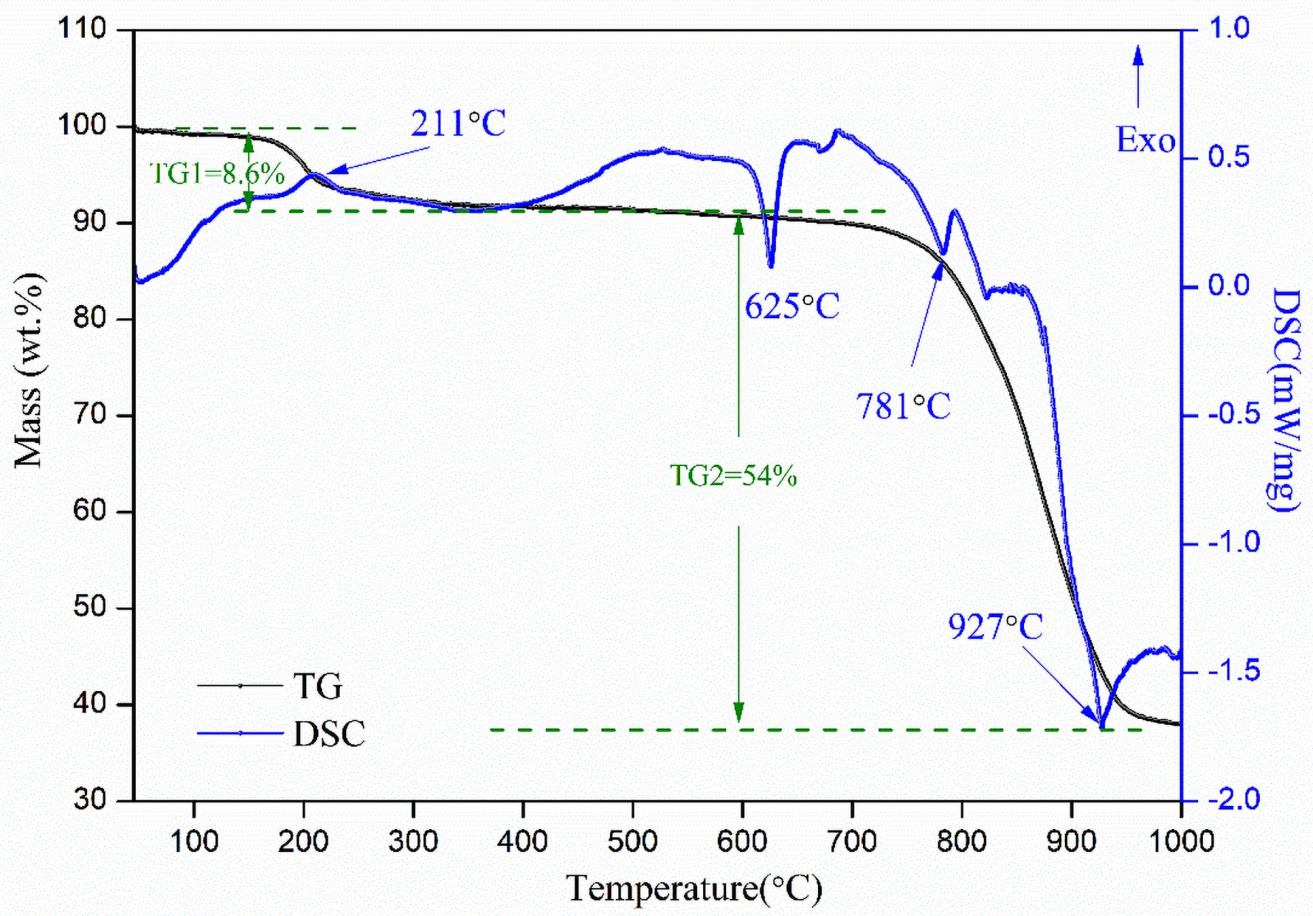
The TG-DSC curve of the industrial waste salt.

TG-DSC experiment has been conducted to study the thermal behavior of the industrial waste salt, and the special specimen with the removal of free water was chosen as the subject. The thermal behavior of the specimen in the temperature ranging from 45 °C to 1000 °C is shown in Fig 3.

It can be seen from the TG-DSC curve that the mass of the specimen presents two obvious weightless processes, which locate in the temperature of 45–600 °C and 600–1000 °C. The first weightless process accounts for 8.6% of the total mass, corresponding to a weak exothermic reaction. Several reactions may contribute to this process, including the decomposition of ammonium salt, evaporation of bound water and the organic impurities pyrolysis etc. The gaseous compounds produced by such reactions are retained in the crucible with the lid (or slowly released form the crucible with the lid), which may react to form new compounds and results in the small exothermic peak around 211°C in the TG-DSC curve [21]. The second process is characterized by rapid weightlessness and the weightlessness is around 54%. The main chemical compositions in the industrial waste salt are NaCl and Na_2_SO_4_, which can be regarded as binary sodium salt. It presents thermal stability and exhibits slow weightless in the temperature below 600 °C. When the temperature exceeds 600°C, the binary sodium salt presents unstable and is broken down to volatilize [22], resulting in the rapid weight loss in TG2 process. The TG2 weightlessness reaction may be made up of three stages i.e. decomposition reaction mainly caused by NaCl, decomposition reaction mainly caused by Na_2_SO_4_ and decomposition reaction mainly caused by NaCl and Na_2_SO_4_. Thus, it results in three endothermic peaks around 625°C, 781°C and 927°C. The thermal behavior of the industrial waste salt indicates that the adopting of heat treatment, such as pyrolysis etc., to remove the organic substance in industrial waste salt may need to control the temperature within 600 °C.

### 3.2 The organic impurity occurrence in industrial waste salt

#### 3.2.1 The organic impurity distribution characteristic

**Fig 4:**
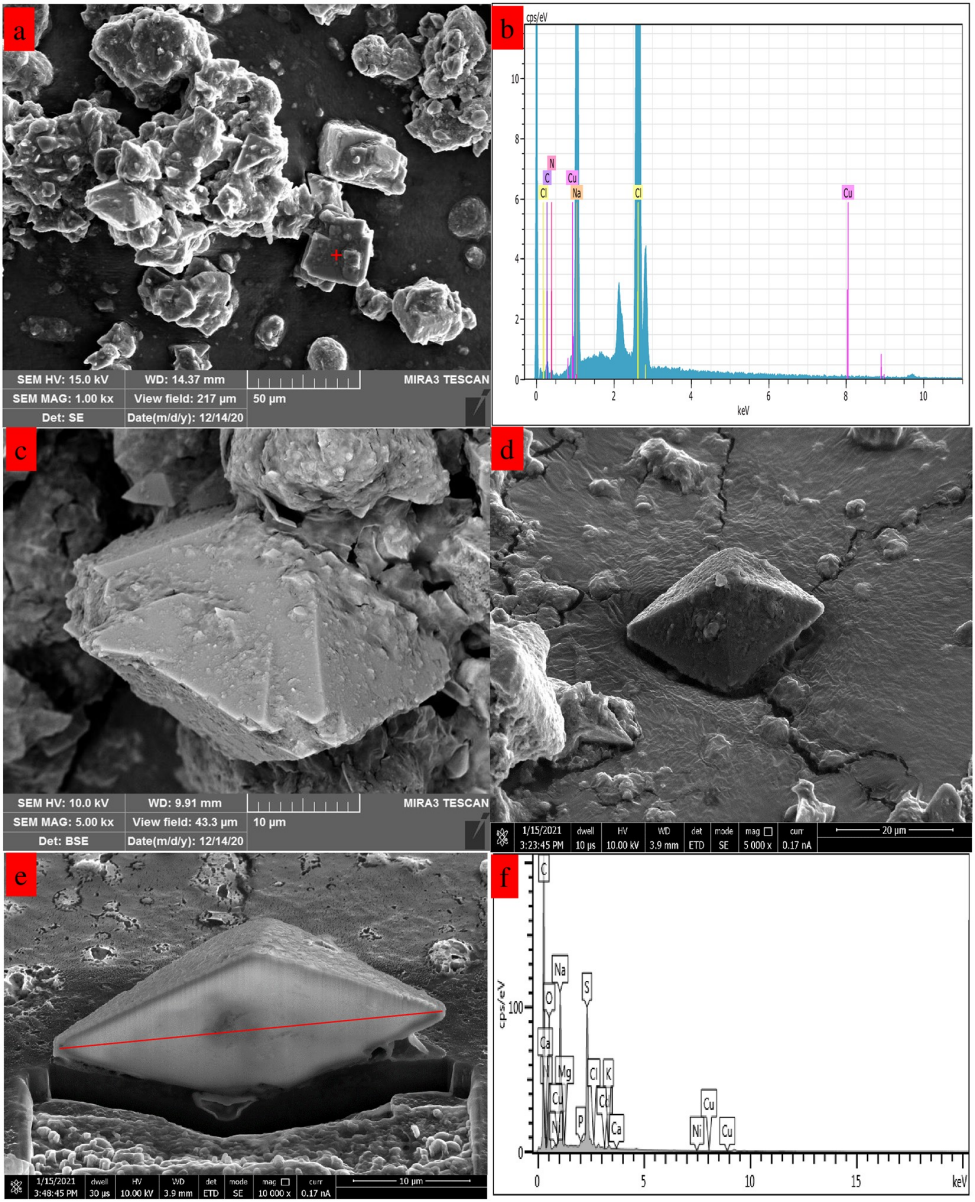
SEM/FIB-SEM for the industrial waste salt.

In order to reveal the organic impurity distribution characteristics in industrial waste salt, a SEM/FIB-SEM experiment was conducted and the results are presented in Fig 4.

It can be seen that the particles with different morphology and size are stacked together. Some particles present regular shape, including cubes and octahedrons; while the irregular particles are adhered to the regular particles. Combination with the XRD results, the regular particles are most possible assigned to NaCl and Na_2_SO_4_ crystals, which are proved by the EDS data results in Fig 4b and 4f, respectively. The impurities exhibit different distribution characteristics in the specimen. Based on the EDS data of Fig 4b and 4f, it can be conclude that the organic impurity, which is represented by carbon element, is distributed on the surface and inside of the particles; while the inorganic impurity (Cu^2+^, Ni^2+^, K^+^ etc.) are mainly distributed in the inner of the particles. During the formation of crystal, the cationic impurities are precipitated in the bulk of the crystal. Thus, it makes the inorganic impurities mainly embedded into the bulk crystal. Besides, the organic impurity may combined with the inorganic impurity in the process of the crystallization process and it will retain the organic impurity in the industrial waste salt.

#### 3.2.2 The organic impurity occurrence characteristic

**Fig 5:**
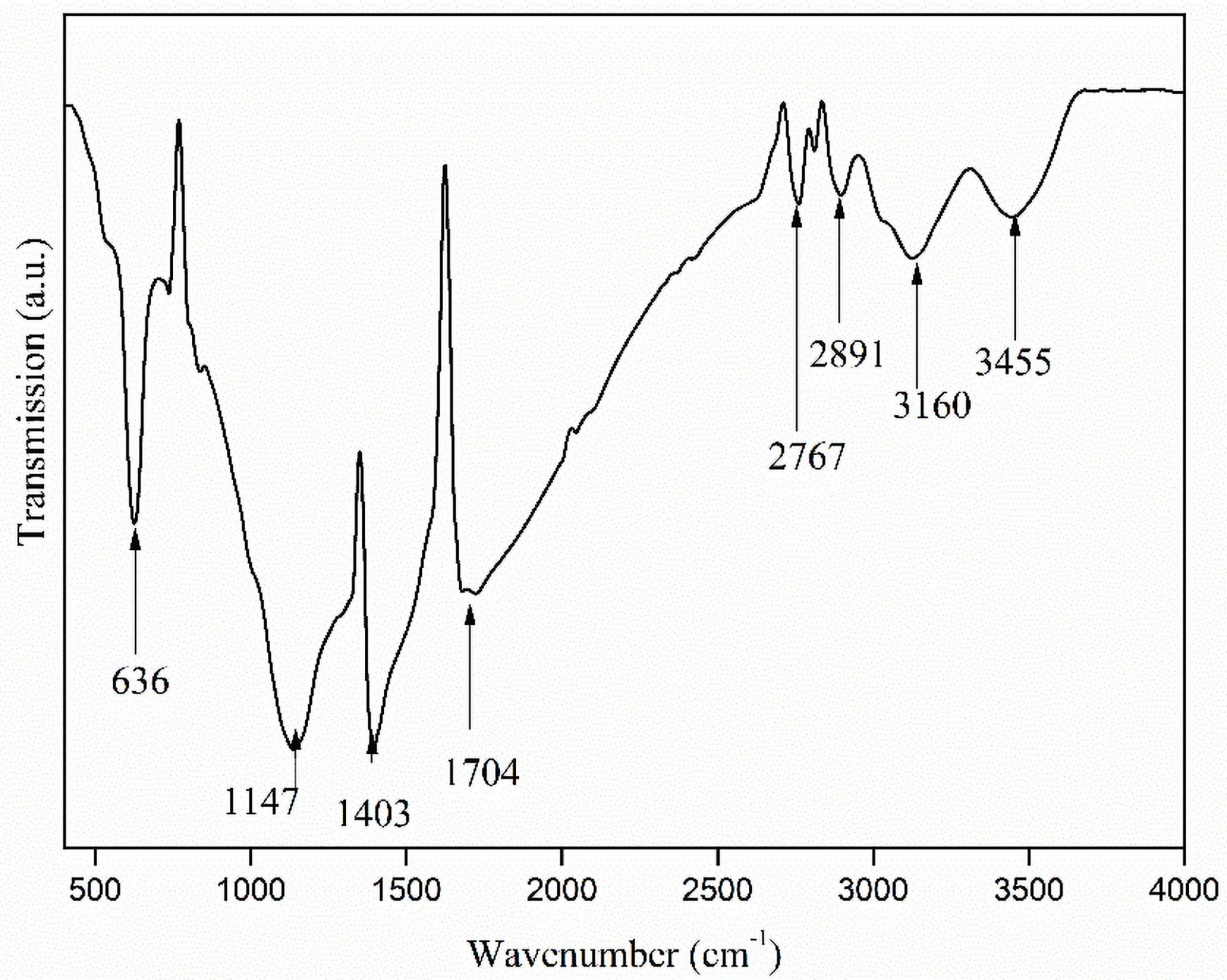
FTIR for the industrial waste salt.

**Fig 6:**
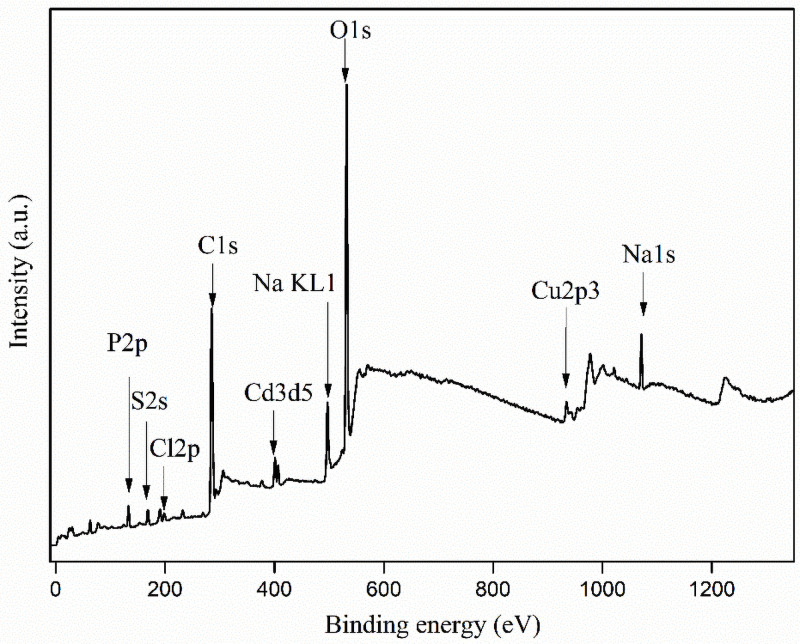
XPS survey spectra for the industrial waste salt.

**Fig 7:**
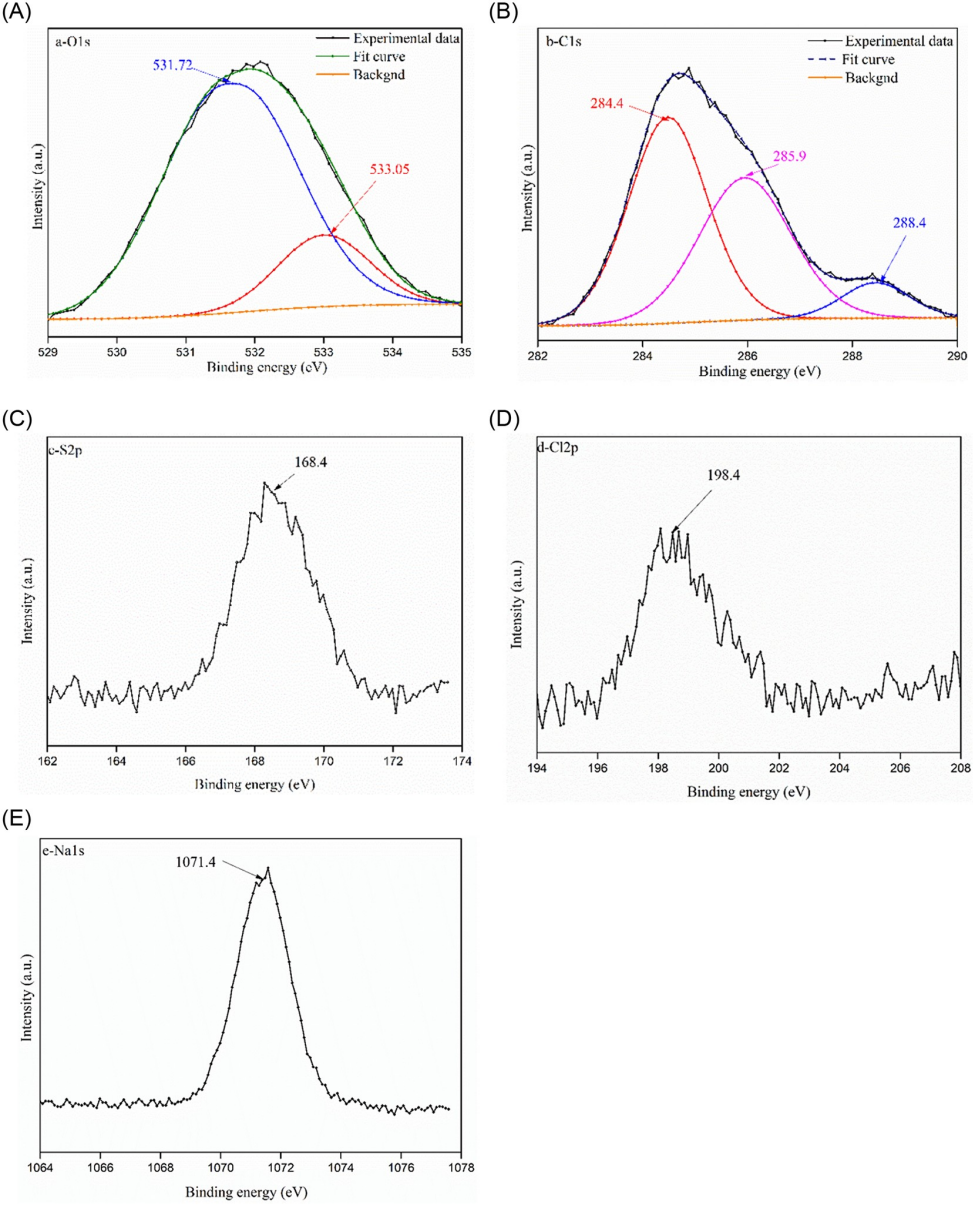
XPS spectra for O1s, C1s, S2s, Cl2p and Na1s.

**Fig 8:**
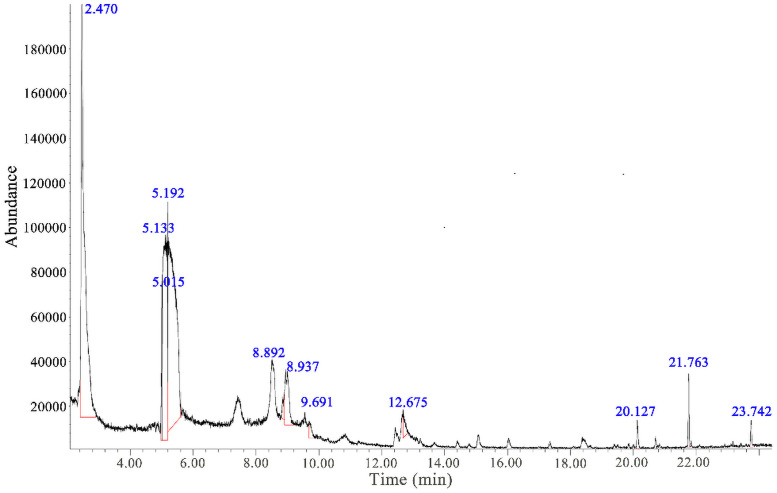
GC-MS detection of organic compounds evolved from the industrial waste salt.

To illustrate the occurrence characteristics of the organic impurity in industrial waste salt, FTIR, XPS and GC-MS were conducted and the results are shown in Figs 5–8.

It can be seen that there mainly exists four strong peaks in the FTIR, which are distributed around 636 cm^-1^, 1147 cm^-1^, 1403 cm^-1^. 1704 cm^-1^. In addition, another four minor weak peaks are also observed around 2767 cm^-1^, 2891 cm^-1^, 3160 cm^-1^, 3461 cm^-1^. The peak located around 636 cm^-1^ is the evidence to the possible existence of formic acid in the industrial waste salt [23]. The FTIR wavenumber ranging from 1033–1160 cm^-1^ is related to C-O/C-O-C groups [24]. Therefore, the FTIR peak of 1147 cm^-1^ in the specimen proves the similar groups in the industrial waste salt. While the FTIR wavenumber around 1350–1450 cm^-1^ is linked to sulfone or sulphate groups (S═O) [25]. Hence, it is reasonable to assign the FTIR peak of 1403 cm^-1^ to sulphate in the specimen. The peak around 1704 cm^-1^ is most likely due to the C = O stretch vibration in ketone [26]. The peak near 2767 cm^-1^ might derives from -NH-HOO-association function group [27]. The small peak around 2891 cm^-1^ is possible due to the C-H stretching in -CH_2_ or -CH_3_ group [28]. The FTIR peak around 3160 cm^-1^ may be assigned to *v*(NH) [29]. The FTIR wavenumber interval of 3450–3470 cm^-1^ is most possible related to phenolic hyreoxyl and aliphatic hydroxide radical [30]. And therefore, the band at 3455 cm^-1^ is possible belonging to this function group. The FTIR analysis above illustrates the existence of complicated organic compounds in industrial waste salt, including lipids and ketones etc. These organics deposit on the surface of industrial waste salt and even combine with metal cations through hydroxyl or carboxyl groups etc., which increases the stability of organic impurity in industrial waste salts.

According to the SEM/FIB-SEM results, the impurities prefer to precipitate on the particles surface in the formation of the industrial waste salt. Therefore, the XPS experiment can be used to further confirm the impurity element distribution feature and the results are shown in Fig 6. The survey spectrum of the analyzed specimen is dominated by the signals of chlorine, sodium, carbon and oxygen elements. Besides, a small content of sulfur, phosphorus, copper and cadmium are also observed. To have a well understand of the impurity properties in the surface, the detailed XPS spectra of O1s, C1s, Cl2p, S2p and Na1s are shown in the Fig 7.

The detailed XPS spectra of the elements can be divided into two categories, i.e. complex chemical state elements that includes O1s, C1s and single chemical form elements, including Cl2p, S2p and Na1s. For the O1s, it mainly contains covalent-combined state oxygen and ionic-combined state oxygen. Therefore, the asymmetric O1s spectrum can be deconvolved into two symmetric sub-peaks at the binding energy of 533.05 eV, 531.72 eV. The peak with the binding energy around 533.05 eV is mainly due to the existence of oxygen-containing complicated organic compounds etc.; while the peak with binding energy near 531.72 eV is assigned to the oxygen-containing inorganic salts etc.; To demonstrate the C-containing compounds in the industrial waste salt, the C1s are deconvolved into three sub-peaks around 284.4 eV, 285.9 eV and 288.4 eV in Fig 7b. The peak around 284.4 eV is most probably due to C-OH/C = O groups [31]; while the peaks with the binding energy of 285.9 eV and 288.4 eV are most probably ascribed to carbonyl group (>C = O) and aliphatic/aromatic carbon group (C–C/CH_x_/C = C) [32].

The XPS spectra of S2p, Cl2p and Na1s present simple chemical environment in the specimen. The S2p with the binding energy at 168.4 eV belongs to sulfates with higher oxidation values [33]. The Cl2p located around 198.4 eV is most probably due to chloride, including NaCl etc., in the specimen [34]. While the Na1s with the binding energy around 1071.43 eV is most probably ascribed to sodium-containing salt, including NaCl, Na_2_SO_4_ etc. The XPS analysis contributes to understand the surface chemical properties of elements and it proves the existence of complex functional group of the organic impurity in industrial waste salts.

To have a clear understand of the organic impurity species, the GC-MS analysis was conducted and the results were presented in Fig 8 and Table 2.

**Table 2:** The parameters and the possible organic compound results from the GC-MS.

Label	Molecular formula	Compound	*t* (min)	Relative area (%)
2.470	C_2_H_4_O	Acetaldehyde	2.470	34.51
5.015	C_17_H_14_N_2_O_2_	Quinazolin-4(3H)-one, 2-[4-(hydrox yphenyl)ethenyl]-3-methyl-	5.015	2.72
5.133	C_6_H_6_Cl_2_N_2_SO_2_	Pyridine-3-sulfonamide, 2, 6-dichloro-4-methyl-	5.133	19.17
5.192	C_8_H_6_N_2_O_4_	p,.beta.-Dinitrostyrene	5.192	32.66
8.892	C_3_H_9_N_3_	Guanidine, N,N-dimethyl-	8.892	0.94
8.937	C_2_H_4_FNO	Acetamide, 2-fluoro-	8.937	4.74
9.691	C_5_H_8_FN_3_	4-Fluorohistamine	9.691	0.96
12.675	C_6_H_18_O_3_Si_3_	Cyclotrisiloxane, hexamethyl-	12.675	1.26
20.127	C_6_Cl_2_D_4_	1,2-Dichlorobenzene-D4	20.127	0.83
21.763	C_21_H_26_F_5_NO_2_Si_2_	Benzeneethanamine, N-[(pentafluoro phenyl)methylene]-.beta.,4-bis[(trimethylsilyl)oxy]-	21.763	1.50
23.742	C_10_H_18_Si	Silane, (2-ethyl-3, 3-dimethyl-4-methylene-1-cyclopenten-1-yl) trimethyl-	23.742	0.71

The GC-MS results directly reveal that there exists nearly 11 kinds of volatile organic compounds, which includes, aldehyde, benzene and its derivatives etc. In addition, the main composition of the organic impurity include the [acetaldehyde], [pyridine-3-sulfonamide, 2, 6-dichloro-4-methyl-] and [p,.beta.-Dinitrostyrene] and their relative content accounting for 86.34% of the total volatile organic compounds in the industrial waste salt. These impurities hold the following properties: the containing of complex functional group structure, such as benzene ring structure, ester group, sulfonamide etc.; owing halogen elements, such as F, Cl. These characteristics make organic impurities have a certain affinity for heavy metals and make the organic impurities owe the thermal stability and non-volatile property in the industrial waste salt.

## 4. Conclusion

(1) The impurities in the industrial waste salt are classified into inorganic and organic impurities. The former include Ni^2+^, Mg^2+^, Cu^2+^ etc. cationic and F^-^, NO^3-^ etc anionic. While the organic impurities are represented in the form of TOC in the industrial waste salt. The industrial waste salt exhibits two weightlessness processes in the TG-DSC curve, i.e. 45–600°C and 600–1000°C. The organic impurity pyrolysis mainly happen within the temperature of 600°C.

(2) The organic impurities distribute both on the surface and inside of the solid salt particles, owing complex function group, such as C-O/C-O-C, C-OH/C = O, C–C/CH_*x*_/C = C etc.; while the inorganic impurities are mainly embedded into the bulk crystal. Meanwhile, the organic substance may combine with metal cations through functional groups, such as hydroxide, carbonyl etc., which increases its stability in the industrial waste salt.

(3) GC-MS analysis has clearly proved that the main organic impurities include 11 kinds of organic compounds in the industrial waste salt, which contains aldehyde, benzene and its derivatives etc. These organic compounds owe the thermal stability and non-volatile property, increasing the difficulty of resource utilization of the industrial waste salt.
